# Bile Nephropathy in Flucloxacillin-Induced Cholestatic Liver Dysfunction

**DOI:** 10.1155/2016/4162674

**Published:** 2016-02-24

**Authors:** Basil Alnasrallah, John F. Collins, L. Jonathan Zwi

**Affiliations:** ^1^Department of Nephrology, Auckland City Hospital, Auckland 1023, New Zealand; ^2^Department of Pathology, Auckland City Hospital, Auckland 1023, New Zealand

## Abstract

Kidney injury in the context of cholestatic liver dysfunction is not uncommon; this has been historically referred to as cholemic nephrosis implying a direct deleterious renal effect of cholemia. However, scepticism about the exact role that bile and its constituents play in this injury has led to the disappearance of the term. We describe a case of severe AKI due to bile nephropathy with bile casts in flucloxacillin-induced liver dysfunction. We also discuss the recent literature reviving the concept of bile nephropathy.

## 1. Introduction

The concept of renal injury in cholestasis was described nearly a century ago by Haessler et al. [1]. Early papers referred to this as cholemic nephrosis with distinctive histological features [2–4]. However, the term has fallen out of favour in recent decades which may reflect doubts about this entity. Cholestatic liver dysfunction is frequently associated with other adverse factors that can cause renal injury such as hypovolemia, endotoxemia, and exposure to other nephrotoxins. Therefore, delineating the negative renal effect of bile and its constituents can be challenging. Furthermore, the previous few decades witnessed an ample interest in hepatorenal syndrome (HRS). The AKI in HRS is largely due to haemodynamic changes and renal hypoperfusion. The clinical diagnosis of HRS might have encompassed many cases where bile was a contributory factor in the AKI. Recent evidence suggests significant overlap between the two [5].

The pathogenesis of bile nephropathy remains a matter of debate which may explain the inconsistent nomenclature used to describe the entity [5–8].

We present a case of bile nephropathy with bile casts in flucloxacillin-induced cholestatic liver dysfunction followed by partial recovery of renal function after resolution of cholemia.

## 2. Case

A 60-year-old man presented with few days of jaundice, generalized fatigue, and dark urine. On admission, he had a bilirubin level of 836 *μ*mol/L (normal < 24), direct bilirubin of >580 *μ*mol/L (normal < 5), ALP of 235 U/L (normal 40–130), GGT of 340 U/L (normal < 60), AST of 236 U/L (normal < 45), ALT of 75 U/L (normal < 45), albumin of 21 g/L (normal 38–52), and serum creatinine of 135 *μ*mol/L (normal 60–105). His creatinine 6 months earlier had been 80 *μ*mol/L.

He had been taking flucloxacillin 500 mg daily intermittently for 3 months to treat skin folliculitis. He stopped the antibiotics 6 weeks prior to his presentation. There was no history of substance or alcohol abuse. He was on no other regular medications.

*On Examination*. He was afebrile and had BMI of 30, BP of 125/80, and heart rate of 70/min. His JVP was +2 cm above the sternal angle, with mild peripheral oedema, clear chest on auscultation, and nontender liver extending 3 cm below the costal margin.

The screen for viral and metabolic causes of hepatitis was unremarkable. An abdominal CT with contrast showed hepatomegaly with diffuse fatty changes but no biliary dilatation. The serum bilirubin continued to rise reaching a peak of 881 *μ*mol/L on day 8. A liver biopsy was performed and showed panacinar steatosis, moderate steatohepatitis (NAFLD score 5/8), portal fibrosis, and cholestasis. An ERCP was normal. The diagnosis was flucloxacillin-induced hepatitis.

The patient's serum creatinine continued to rise and peaked at 581 *μ*mol/L on day 16 despite adequate hydration and avoidance of nephrotoxins. In addition, the patient had persistent severe hypokalemia with serum potassium levels consistently <3 mmol/L despite the potassium replacement. Arterial blood gas was not suggestive of renal tubular acidosis with serum bicarbonate level of 26 mmol/L and pH of 7.46.

On day 3, his urine sodium was 33 mmol/L, urine potassium 42 mmol/L, and urine osmolality 420 mmol/kg. The transtubular potassium gradient was >10 and the serum potassium at the time of specimen collection was 2.7 mmol/L indicating renal cause for his hypokalemia. The urinary protein/creatinine ratio was slightly raised at 39 mg/mmol (normal < 23) and otherwise bland urine sediment. The urine output averaged 1.5 L per day from the day of admission.

A renal USS showed normal sized kidneys at 128 mm and 125 mm with no obstruction, normal echotexture, and perfusion; a renal biopsy was performed.

## 3. Renal Biopsy

**Figure 1:**
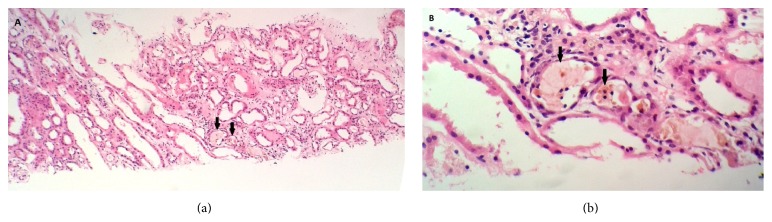
(a) Multiple tubules with dilatation and vacuolization. Tubular lumens demonstrate heavily pigmented granular casts (black arrows). (b) Multiple densely stained cellular and granular casts in the tubular lumen (black arrows) (haematoxylin and eosin stain).

Glomeruli were normal. The interstitium showed mild diffuse oedema and minimal infiltration by chronic inflammatory cells. Most tubules were dilated, a few containing necrotic tubular cells (Figure 1(a)), some with yellow/brown colouration. A few acellular proteinaceous solid brown casts were present (Figure 1(b)). Tubular epithelium showed evidence of injury, including desquamation and necrosis. A few tubules showed disruption of the entire luminal border, with yellow granules in tubular cell cytoplasm. Immunofluorescence and electron microscopy showed no abnormalities. The biopsy was consistent with acute tubular necrosis with bile staining and bile casts.

## 4. Progress

The patient's bilirubin began to improve on day 10 and a week later the serum creatinine also started to fall. On day 75, the bilirubin was 50 *μ*mol/L and the serum creatinine was 164 *μ*mol/L (Table 1).

## 5. Discussion

We have described a case of acute nonoliguric kidney injury characterized by potassium wasting in a patient with cholestatic liver dysfunction secondary to flucloxacillin. Both older age (>55 years) and a prolonged duration of flucloxacillin treatment (>14 days) are known risk factors for liver injury which can happen many weeks after stopping the antibiotics [9]. The renal biopsy findings and clinical features were consistent with previous descriptions of bile nephropathy.

Functional causes of renal insufficiency such as hepatorenal syndrome (HRS) were excluded by the urinary sodium >20 mmol/L, nonoliguria, and features of ATN seen on the renal biopsy [10]. In a recent study, Van Slambrouck et al. found that 24 out of 44 jaundiced patients had renal bile casts on biopsy ranging from mild to severe depending on the extent of nephron involvement [5]. Moreover, 85% of the patients with HRS had renal bile casts, whereas only 42% patients without HRS had bile casts. Patients with bile casts had significantly higher levels of serum total bilirubin and conjugated bilirubin than those who did not. Also, 73% of the biopsies showed variable degrees of ATN, similar to the finding in our patient. It has been proposed that the bile casts may have a direct tubular toxic effect and an obstructive effect similar to that of myeloma cast nephropathy. However, Heyman et al. pointed out in a commentary to the previous paper that the presence of bile casts might merely reflect reduced glomerular filtration rate (GFR) through a reduction in the washout of the casts without an actual role in the pathogenesis [11].

Nevertheless, bile and its constituents have been reported to exert toxic effects on the renal tubules which can result in proximal tubular dysfunction [12]. The severe potassium wasting in this patient can be consistent with this type of tubular injury. Unfortunately, other urinary markers for proximal tubular dysfunction were not available, for example, glucosuria and phosphaturia.

There have been attempts to identify the culprit in bile nephropathy. Recent experimental evidence strongly suggested that bile acids are the cause of the renal injury in common bile duct ligated (CBDL) mice. Prefeeding the mice with norUrsodeoxycholic acid (a hydrophilic bile acid much less toxic than physiological bile acids) for 7 days before CBDL prevented tubular epithelial injury at 3 days [13]. The authors propose that the tubular injury by bile acids is accompanied by basement membrane disruption which leads to the leak of bile acid rich urine into the renal parenchyma, leading to further damage, interstitial inflammation, and fibrosis. However, renal effects of cholestasis can vary widely across different species, being even renoprotective in some [14, 15]. Therefore, extrapolating these results to humans might be too speculative.

Bilirubin has also been implicated as a potential mediator of this injury. However, its role is also controversial with both deleterious and advantageous renal effects being reported in the literature. Nazar et al. found that, in AKI in hepatic failure, terlipressin was effective in only 13% of patients with serum bilirubin >10 mg/dL compared with 67% of patients with lower bilirubin [16]. The nephrotoxic mechanism appears to be inflammatory as it is attenuated by the use of sirolimus [17].

Other studies reported hyperbilirubinemia to be renoprotective in both animals and humans through anti-inflammatory and antioxidative effects [18–20]. Guo et al. have shown that higher bilirubin levels were independently associated with lower levels of late graft failure in a prospective study in renal transplant recipients [14]. The disparity between these findings might be due to different effects of hyperbilirubinemia in different models of disease such as concurrent ischemia, reduced GFR, or other associated tubular conditions [20].

It is worth noting that cholestasis affects renal function adversely in indirect ways too. Cholemia alters vascular hemodynamics by impairing endothelial vasoactivity and diminishing response to catecholamines. Furthermore, cholestasis is accompanied by the lack of bile in the intestine which increases gut permeability and bacterial translocation [21]. Uslu et al. tried to minimize the effect of these factors through meticulous attention to fluid status, electrolytes balance, and prophylactic antibiotics in a prospective study of patients with obstructive cholestasis. Despite that, ATN and venous dilatation on the renal biopsies were universal (100% in 20 patients) which suggest renal injury despite these measures.

## 6. Summary

There are a variety of mechanisms mediating AKI in patients with cholestatic liver dysfunction. Clinical and animal research evidence show that high levels of filtered bile lead to tubular toxicity and bile cast formation, which may initiate and/or propagate renal injury. The term bile nephropathy encompasses all the adverse effects that bile and its constituents have on the kidneys. The case of bile nephropathy with bile casts that we described is an extreme end of the spectrum. The lack of bile casts in the renal biopsies of jaundiced patients should not discount the diagnosis of bile nephropathy. This case highlights the need to take account of the consequences of high bile levels in the pathogenesis of AKI in in the context of acute liver disease.

## Figures and Tables

**Table 1:** Pertinent laboratory data.

Laboratory values	Day 1	Day 8	Day 16	Day 24	Day 30 (D/C)	Day 75
Total bilirubin (*µ*mol/L)	836	**881**	664	472	312	50
Scr (*µ*mol/L)	135	361	**581**	523	377	164
SU (mmol/L)	9.4	16.7	28	32		13.7
Serum K (mmol/L)	2.9	3.6	4.7	3.5	3.5	4.2
ALP (U/L)	235	188	200	134	137	108
GGT (U/L)	340	155	119	43	41	113
AST (U/L)	234	240	228	146	137	42
ALT (U/L)	75	128	131	90	86	
Alb (g/L)	21	34	28	32	29	33

Note: conversion factors for units: bilirubin in *µ*mol/L to mg/dL *∗* .0585; Scr in *µ*mol/L to mg/dL *∗* .0113; SU in mmol/L to mg/dL *∗* 2.8011.

Scr: serum creatinine; SU: serum urea; ALP: alkaline phosphatase; GGT: gamma-glutamyl transferase; AST: aspartate transaminase; ALT: alanine transaminase; Alb: albumin.
